# Establishing Murine Intestinal Organoids to Study Nutrient- and Tastant-Evoked Gut Signaling

**DOI:** 10.3390/nu18121995

**Published:** 2026-06-19

**Authors:** Praveen Kumar, Florian Ziegler, Maik Behrens

**Affiliations:** Leibniz Institute for Food Systems Biology at the Technical University of Munich, 85354 Freising, Germany; p.kumar.leibniz-lsb@tum.de (P.K.);

**Keywords:** murine intestinal organoids, enteroendocrine cells, chromogranin A, cholecystokinin, L-glutamic acid, denatonium benzoate

## Abstract

**Background/Objectives**: Numerous studies have investigated the responses of the gastrointestinal tract to tastants, particularly in specialized enteroendocrine and other chemosensory cells. However, many of these investigations used various taste stimuli often at high concentrations or relied on immortalized cell lines or heterogeneous cell populations, which can limit their physiological relevance and reproducibility. To establish a stable, physiologically representative model system for consistently investigating gut epithelial responses to tastants, our study developed 3D murine intestinal organoids (MIOs). **Methods**: Murine intestinal organoids were generated from isolated intestinal crypts and cultured under defined conditions to maintain epithelial differentiation. Organoids were stimulated with selected nutrients and tastants, and downstream signaling responses were assessed using hormone secretion assays. **Results**: The 3D MIO culture system was successfully established, providing a robust in vitro platform for studying extraoral bitter sensing and release of the enteroendocrine hormone cholecystokinin. Moreover, 5 mM denatonium benzoate and 30 mM L-glutamic acid specifically induced cholecystokinin secretion in MIOs, whereas other bitter or non-bitter stimuli did not. **Conclusions**: Murine intestinal organoids provide a stable model for studying nutrient- and tastant-evoked signaling in the gut. This approach enables precise investigation of underlying mechanisms and may advance our understanding of gut chemosensation and metabolic regulation.

## 1. Introduction

Bitterness is a fundamental taste, akin to sweet, salty, sour, and umami, and is frequently perceived as sharp, harsh, or disagreeable; however, some individuals appreciate it in foods such as coffee or dark chocolate [1,2]. Humans and animals utilize the bitter taste primarily to identify pharmacologically active substances, which include poisonous as well as beneficial substances, such as medicines or vitamins. Moreover, they vary in the quantity of bitter receptors, sensitivity levels, and behavioral responses [2,3].

Bitter taste receptors in humans (~25 subtypes) and in mice (~35 subtypes), called TAS2Rs and Tas2rs (Taste 2 receptors) respectively, belong to the G protein-coupled receptor (GPCR) gene family [4,5,6]. They show a defined expression pattern in taste papillae and a more selective, lower-level expression in extraoral tissues [7,8,9,10]. In addition, they are expressed in several intestinal cell types, including enteroendocrine cells [11,12], tuft cells [13], Paneth cells [10,14], and certain goblet cell populations [9].

Like humans, mice show distinct agonist activation profiles due to differences in tuning breadth, ortholog specificity, and sensitivity [8]. Multiple Tas2r genes, such as Tas2r108, Tas2r126, Tas2r135, Tas2r137, and Tas2r143, are expressed at the highest levels across the mouse gastrointestinal tract, whereas Tas2r109 and Tas2r115 are specifically restricted to the stomach and Tas2r117, Tas2r125, and Tas2r131 are specifically enriched in the colon [10]. This spatial pattern indicates functional specialization of bitter sensing along the gut. Mouse intestinal organoids provide a physiologically relevant in vitro system for studying gut chemosensation because they retain the epithelial cell diversity and signaling pathways present in the native intestine. They are particularly valuable for examining how bitter tastants activate enteroendocrine cells to release hormones like cholecystokinin (CCK), glucagon-like peptide-1 (GLP-1), and peptide YY (PYY) [11,15,16]. CCK is one of the key hormones involved in digestive regulation, satiety, metabolic balance, and the detection of potentially toxic dietary compounds. Secreted CCK acts paracrine/autocrine on nearby cells or enters circulation to bind CCK1R/CCK2R (Gq-coupled) on vagal afferents, gallbladder, pancreas, and gastric smooth muscle. This activates PLCβ-DAG-IP_3_, raising Ca^2+^ to promote satiety, bile release, enzyme secretion, and slowed gastric emptying [17,18,19].

In the present study, we established the MIO culture in a controlled 3D model and characterized it by stimulating the differentiated mouse organoids with tastants. This approach provides insights into GI sensing beyond studies performed in GI derived cell lines. Moreover, as not all nutrient-sensing processes in the gut rely on tastants and taste-like signaling, organoids can help distinguish taste receptor-mediated mechanisms from more general food components such as, e.g., lipids or proteins, the prime stimuli for CCK secretion (for a review see [20]). Therefore, this research bridges gaps in tastant-mediated enteroendocrine function, potentially informing therapeutics for gut disorders or food palatability.

## 2. Materials and Methods

Materials: Cucurbitacin E, cycloheximide, denatonium benzoate, dimethyl sulfoxide, D-glucose, famotidine, D-fructose, L-glutamic acid, glycocholic acid, inositol monophosphate (IMP), and linolenic acid were purchased from Sigma-Aldrich (Merck KGaA, Darmstadt, Germany). A cholecystokinin ELISA kit was ordered from Phoenix Pharmaceutical (Karlsruhe, Germany).

Animals: The Rath group at the Technical University of Munich, Chair of Nutrition and Immunology, generously provided the frozen crypts for the cultivation of mouse intestinal organoids. Briefly, the crypts were obtained from 8–18-week-old C57BL/6 mice, which were sacrificed by CO_2_, before the small intestine was removed [21], following established protocols [22,23]. All animals were maintained under specific pathogen-free conditions within the institutional animal facility (ZIEL, Institute for Food and Health, Freising, Germany). All animal procedures were approved by the Bavarian Animal Care and Use Committee.

Generation of organoids and culture: The crypts made available to us were stored in liquid nitrogen. For thawing, one vial was removed from liquid nitrogen and 1 mL of fresh medium (Advanced DMEM/F-12, Thermo Fisher Scientific, Waltham, MA, USA) was added and spun down at 300× *g* for 5 min at 4 °C. The pellet was resuspended in Cultrex basement membrane extract (Bio-Techne, Minneapolis, MN, USA) and plated 15 µL/well (2–3 wells) in 48-well plates. After polymerization of Cultrex, 250 µL of IntestiCult (Stemcell Technologies, Cologne, Germany) containing 1 µM ROCK inhibitor (Y-27632) was added, and the plate was incubated at 37 °C with 5% CO_2_. After 2 days, this medium was replaced by fresh IntestiCult. Every four days, the entire medium was replaced. For passaging, organoids were taken out from Cultrex and mechanically separated into single-crypt domains before being moved to new Cultrex. Every one to two weeks, passaging was carried out using a 1:5 split ratio.

To simulate the organoids with tastants as well as to perform immunohistochemical experiments, we differentiated them by using complete culture medium or CCM (Advanced DMEM/F12 contains the supplements: 1 M HEPES Buffer (PAN Biotech, Aidenbach, Germany), GlutaMAX supplement (Fisher Scientific), penicillin/streptomycin, B-27 supplement (Fisher Scientific), N-2 supplement (Fisher Scientific), N-Acetylcystein (Merck KGaA, Darmstadt, Germany), 10–50 ng ml^−1^ EGF (Immunotools, Friesoythe, Germany), 100 ng ml^−1^ Noggin (Peprotech, Thermo Fisher Scientific, Waltham, MA, USA), 500 ng ml^−1^ R-spondin 1 (Stemcell), and (Z)-4-Hydroxytamoxifen (Peprotech).

Organoid stimulation with tastants: Organoids were cultured and differentiated in 48-well plates at a density of approximately 50 organoids per well using complete culture medium (CCM). Prior to stimulation, organoids were washed three times with HEPES-buffered saline (pH 7.4) composed of 138 mM NaCl, 10 mM HEPES, 4.5 mM KCl, 4.2 mM NaHCO_3_, 2.6 mM CaCl_2_, 1.2 mM NaH_2_PO_4_, and 1.2 mM MgCl_2_. Following washing, organoids were pre-incubated for 30 min at 37 °C in a 5% CO_2_ atmosphere in HEPES-buffered saline supplemented with a DPPIV inhibitor (DPP4, Merck, Lot# 4191671). Subsequently, tastants were added at concentrations defined in prior studies [8,24,25,26], and organoids were incubated for an additional 2 h under the same conditions. The reaction was terminated by placing the plates on ice. Conditioned medium containing secreted factors was collected into microcentrifuge tubes and centrifuged at 400× *g* for 5 min at 4 °C. The resulting supernatant was immediately snap-frozen in liquid nitrogen and stored at −80 °C until further analysis by ELISA.

Cholecystokinin ELISA: CCK release in supernatants collected after tastant stimulation of organoids was quantified using a cholecystokinin (CCK) (26–33), non-sulfated chemiluminescent EIA kit (Phoenix Pharmaceutical, Karlsruhe, Germany, catalog # CEK-069-04) according to the manufacturer’s general workflow. Briefly, 50 µL of standards, controls, or samples were added per well, followed by 25 µL of primary antibody and 25 µL of biotinylated peptide. The plate was incubated at room temperature for 2 h, washed four times with 350 µL/well of 1× assay buffer, and then treated with 100 µL/well streptavidin-HRP solution for 1 h at room temperature. After four washing steps, 100 µL/well TMB substrate was added and allowed to develop for 1 h at room temperature. The reaction was stopped with 100 µL/well of 2 N HCl, and absorbance was measured at 450 nm. CCK concentrations were calculated from the standard curve.

Cryosectioning: Organoids were maintained in CCM for 7 days prior to processing. For harvesting, the culture medium was carefully aspirated and replaced with ice-cold PBS (e.g., 330 µL per well of a 48-well plate). Matrigel domes were mechanically disrupted by repeated pipetting, and the released organoids were transferred into 1.5 mL Eppendorf tubes. Samples were centrifuged at 300× *g* for 5 min at 4 °C, after which the supernatant was gently removed. The pellet was fixated by adding freshly prepared 4% (*w*/*v*) paraformaldehyde in PBS and incubating for 15 min at 4 °C. Following fixation, organoids were pelleted again under the same centrifugation conditions. The fixed organoids were washed three times with PBS and then incubated in 30% sucrose in PBS at 4 °C for 2 h for cryoprotection. Excess sucrose solution was removed, and the organoid pellet was resuspended in a minimal volume of Tissue-Tek embedding medium and stored at −20 °C. Cryosections of approximately 10 μm thickness were prepared using a cryostat, mounted onto microscope slides, and stored at −20 °C until further staining procedures.

Immunohistochemistry: The cryosections of organoids were stained for GLP-1, Anti-chromogranin A, villin, mucin-2, and lysozyme C (Table 1) using the protocols described in [9,10]. Briefly, the sections were fixated in 4% PFA in 1x PBS for 5 min, then washed 3 times with PBS for 5 min each. Except for GLP-1, we performed antigen retrieval with 10 mM sodium citrate (pH 6) for 10 min at −80 °C, then blocked with 5% normal horse serum (with 0.5% Triton X-100 in 1× PBS, 1 h). The blocking was followed by incubation with the primary antibodies (anti-GLP-1, anti-chromogranin A, anti-villin, anti-mucin-2, anti-lysozyme C) diluted in 5% normal horse serum (0.2% Triton X-100 in 1× PBS) overnight at 4 °C. The next day, sections were washed three times for 10 min with PBS, then secondary antibodies were applied (AlexaFluor 488-conjugated; 1:2000) diluted in 5% normal horse serum (0.2% Triton X-100 in 1× PBS) for 1 h at 4 °C. Again, three washes with PBS for 10 min were performed, then nuclei were stained with DAPI (1:1000; in PBS), followed by washing with PBS two times for 5 min. Finally, sections were covered with coverslips using an aqueous anti-fade mounting medium. A Zeiss LSM 780 device (Carl Zeiss AG, Munich, Germany) was used to obtain immunofluorescence images via confocal laser scanning microscopy.

Recording and calculations: For MIO stimulation, an effective concentration of tastant was selected based on prior dose–response data [8,24,25,26] and applied to the organoids. Tastants were diluted in HEPES-buffered saline to maintain physiological pH and osmolarity during stimulation. For ELISA-based hormone measurements, at least three independent experiments were carried out, with duplicate wells used for each tastant concentration to ensure reproducibility. Summary data are presented as mean values with standard error of the mean (SEM). The mean peak response in the luminescence-based assay was compared with the pre-stimulation baseline, and statistical significance was defined as *p* ≤ 0.05, assessed using Student’s *t*-test.

## 3. Results

### 3.1. Establishment of MIO Culture

Analogous to a variety of organoid culture systems that have been described [17,18,19,22], we attempted to establish one for the study of tastant and nutrient sensing in the mouse GI tract. To differentiate the MIO, we used growth supplements such as R-spondin (required for crypt proliferation), epidermal growth factor (EGF; associated with intestinal proliferation), Noggin (to expand the crypt numbers), etc. Cultrex UltiMatrix RGF BME was used to support MIO growth while maintaining basic crypt-villus physiology. The passaging took place every 7–10 days.

**Figure 1:**
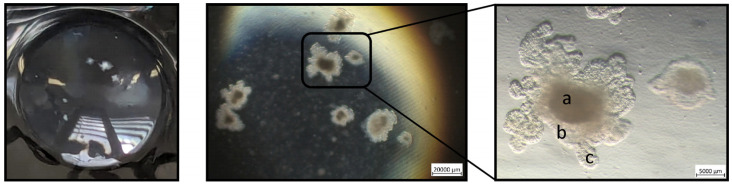
Mouse intestinal organoids. Schematic representation of Cultrex UltiMatrix RGF BME/organoid mixture in the center of a well in a 48-well plate, along with 20,000 µm and 5000 µm zoom. These differentiated mouse intestinal organoids were cultured for one week. a—luminus, b—villus-like structure, c—crypt-like structure.

As evident in Figure 1, MIO cultures grow rapidly and develop 3D organoids with luminal, villus-like, and crypt-like regions.

### 3.2. Differentiation of MIO in All Cell Types

Immunostaining with antibodies against specific cell-type markers confirms organoid differentiation by revealing epithelial polarization, proliferation, and mature cell lineages like enterocytes, goblet cells, and Paneth cells. To visualize successful MIO differentiation, villin, mucin-2, chromogranin A, GLP-1, and lysozyme were used as markers (Table 2). The presence of mature cell types was confirmed by immunohistochemical staining for each marker protein.

**Figure 2:**
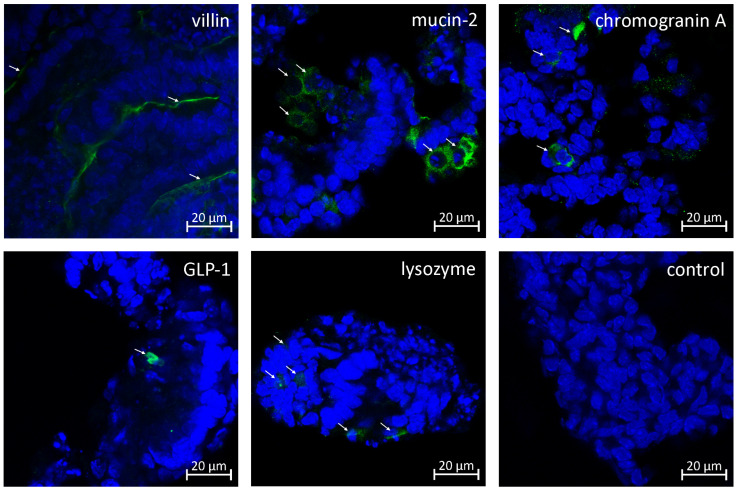
Immunohistochemical staining of MIO derived from a single stem cell. To label different cell types in MIO, cryosections of differentiated organoids were fixed with 4% PFA. Then, sections were stained with antibodies against villin, mucin-2, chromogranin A, GLP-1, and lysozyme to demonstrate enterocytes, goblet cells, enteroendocrine cells, L-type enteroendocrine cells, and Paneth cells, respectively. Fluorescence signals were obtained by using AlexaFluor 488-conjugated secondary antibodies (green) and with DAPI (blue). Experiments without primary antisera served as negative controls. Scale bars are represented at the bottom of each image, and arrows indicate positive signals.

As demonstrated in Figure 2, immunohistochemical staining experiments of MIO confirmed the presence of enterocytes, as evident from the staining of the brush border membrane by the anti-villin antiserum. The primary functions of enterocytes are absorption of nutrients and water, forming tight junctions for barrier and antigen presentation. The anti-mucin-2 antiserum revealed the existence of goblet cells in the MIO. Goblet cells secrete mucins that form the mucus layer, which protects against microbes. The anti-lysozyme antiserum labeled the Paneth cells. These cells secrete antimicrobial peptides (e.g., defensins and lysozyme) and support the stem cell niche. The anti-chromogranin A antiserum documents the existence of enteroendocrine cells, which form several lineages of which the enteroendocrine L-cells are known to produce and secrete the peptide GLP-1 highlighted by the anti-GLP-1 antiserum. These cells sense nutrients and secrete hormones (e.g., GLP-1, CCK) that regulate motility and metabolism [27,28,29,30].

### 3.3. MIO Secretion of CCK upon Stimulation with Tastants Attests to the Function of Differentiated Organoids

**Figure 3:**
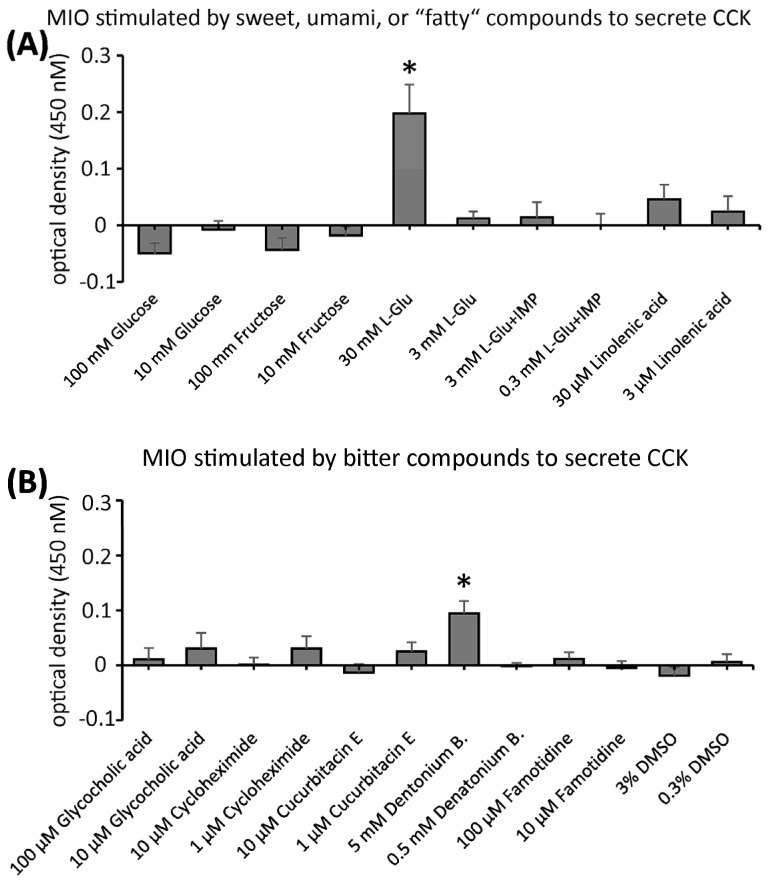
CCK secretion by MIO in the presence of sweet, umami, fatty, and bitter compounds. The MIOs are cultured and differentiated in CCM for one week. (**A**) MIO was stimulated by sweet, umami, and fatty compounds with two different concentrations. A significant increase in CCK secretion was observed in the presence of 30 mM L-glutamic acid. (**B**) MIO was stimulated by bitter substances with two different concentrations. A significant increase in CCK secretion was observed in the presence of 5 mM denatonium benzoate. The y-axis shows the optical density at 450 nm, and the x-axis lists the different tastants. Data were collected from at least three independent experiments performed in at least duplicates (*n* = 3, two technical replicates each). Data represent means ± standard error of mean (SEM). Asterisks indicate statistical significance (*p* ≤ 0.05, Student’s *t*-test).

To determine whether enteroendocrine cells of MIOs respond to tastant stimulation with CCK release, a battery of stimuli was applied to the organoids at different concentrations and the conditioned supernatants were analyzed for CCK by ELISA (Figure 3). As stimuli, D-glucose (100 mM, 10 mM), fructose (100 mM, 10 mM), L-glutamic acid (30 mM, 3 mM), 0.2 mM inositol monophosphate with L-glutamic acid (3 mM, 0.3 mM), linolenic acid (30 µM, 3 µM), glycocholic acid (100 µM, 10 µM), cycloheximide (10 µM, 1µM), cucurbitacin E (10 µM, 1 µM), denatonium benzoate (5 mM, 0.5 mM), and famotidine (100 µM, 10 µM) were used.

Upon stimulation with taste compounds, CCK rises slightly but significantly when stimulated with 30 mM L-glutamic acid (CCK-concentration = 0.0671 ± 0.0537 pg/mL) and 5 mM denatonium benzoate (CCK-concentration = 0.0026 ± 0.0013 pg/mL).

According to previous findings obtained in cell-based in vitro assays, high concentrations (100 mM) of glucose or fructose strongly stimulate intestinal hormone secretion in mouse models, primarily by promoting metabolism in enteroendocrine L-cells, leading to ATP production and hormone exocytosis [31,32]. Glucose potentially stimulates both GLP-1 (from L-cells) and GIP (from K-cells) secretion, while fructose selectively triggers GLP-1 but not GIP in mice, as seen in gavage studies [33]. In our study, we observed no CCK secretion by MIO in the presence of sweet substances (glucose and fructose), consistent with previous findings.

Umami stimuli such as L-glutamate, especially when combined with IMP, activate the Tas1r1/Tas1r3 receptor (expressed in CCK-positive I cells) and increase CCK secretion in STC-1 cells [34,35]. In our study, we found that 30 mM L-glutamic acid induced CCK secretion, whereas 3 mM was insufficient, even after adding IMP. There is also evidence that long- and medium-chain fatty acids (including alpha-linolenic acid and related compounds) act on nutrient-sensing receptors on enteroendocrine L-cells, thereby driving GLP-1 and PYY release [36,37]. In addition, similar to other bile acids, glycocholic acid can enhance GLP-1 secretion [38,39,40]. In our study, we used the fatty stimulus linolenic acid [24] (30 µM and 3 µM) and the bitter stimulus glycocholic acid [26] (100 µM and 10 µM) to stimulate MIO for CCK secretion, but observed no enhancement. As further bitter compounds, we chose cycloheximide [5] (10 µM, 1 µM), cucurbitacin E (10 µM, 1 µM), denatonium benzoate (5 mM, 0.5 mM), and famotidine (100 µM, 10 µM) [8] to stimulate MIO. According to our results, 5 mM denatonium benzoate induces CCK secretion in MIOs, whereas other stimuli do not. This suggests that CCK release depends on ligand-specific activation of bitter receptor signaling rather than a generic response to bitterness.

## 4. Discussion

The mouse organoids, compared with human organoids, are often preferred for mechanistic studies tied to genetics, while human organoids are essential for translational relevance [41,42]. Mouse intestinal organoids are generally more advantageous than isolated mouse intestinal cells for studying hormone secretion because they preserve 3D tissue architecture, maintain multiple epithelial cell types, and more faithfully preserve key niche-dependent functions, such as nutrient sensing and incretin release, than flat cell cultures. They also allow us to test how stem cells differentiate into hormone-producing enteroendocrine cells in a controlled but more physiologic system [21,41,43,44]. The main interpretation is that mouse intestinal organoids can act as a model for luminal chemosensation: they help show how bitter compounds may alert the epithelium to toxins, parasites, or other stressors and then trigger protective responses. This is why these organoid studies are often discussed in the context of gut immunity, epithelial defense, and hormone secretion rather than flavor perception. The evidence is still incomplete, but the direction is clear: bitter substances can be biological signals in the mouse intestine, not just unpleasant tastes [11,44,45].

In our study, MIO culture’s demonstration of CCK release in response to L-glutamic acid and denatonium benzoate stimulation validates functional bitter taste receptor (TAS2R/Tas2r) signaling in differentiated enteroendocrine cells within organoids. This bridges oral taste mechanisms to gut nutrient sensing, offering a scalable 3D model for dissecting agonist-specific responses beyond 2D cell lines like STC-1.

Denatonium benzoate, a canonical Tas2r agonist, triggers Ca^2+^ mobilization via gustducin and voltage-sensitive channels, culminating in CCK secretion from enteroendocrine cells, as replicated here in intact MIOs [46,47,48,49]. L-glutamic acid’s effect suggests umami/bitter crosstalk through Tas1r1/Tas1r3 or CaSR pathways, mirroring prior reports of amino acid-induced CCK in mouse gut tissue [35,50].

Our findings extend the emerging concept that bitter taste receptors in the gut function as nutrient and toxin sensors. The 3D MIO system recapitulates key aspects of intestinal chemosensation by demonstrating that only specific stimuli—5 mM denatonium benzoate and 30 mM L-glutamic acid—elicit CCK secretion, whereas other bitter or non-bitter compounds do not, indicating that enteroendocrine responses are ligand-selective rather than generic “tastant” detectors. This supports the idea that different bitter ligands engage distinct receptor combinations or signaling thresholds, which may underlie heterogeneous physiological outcomes such as satiety modulation, bile release, or epithelial defense, and highlights the importance of using 3D organoid models to retain physiologically relevant receptor coupling and Ca^2+^ dynamics that are often lost in 2D systems. In the very comprehensive work of Lossow and colleagues, 34 mouse Tas2rs were matched with over one hundred chemically diverse cognate bitter substances [8]. The activation of mouse Tas2rs by the bitter bile acid glycocholic acid, amongst others, was investigated by Ziegler and colleagues [26]. According to these studies, our CCK secretion experiment employed bitter tastants for the receptors Tas2r105 (cycloheximide, cucurbitacin E, denatonium benzoate, glycocholic acid), Tas2r108 (glycocholic acid), Tas2r114 (cucurbitacin E), Tas2r117 (glycocholic acid) Tas2r123 (denatonium benzoate, glycocholic acid), Tas2r126 (glycocholic acid), Tas2r135 (denatonium benzoate), Tas2r140 (denatonium benzoate), and Tas2r144 (denatonium benzoate, glycocholic acid) (Table 3). Of these receptors a comprehensive expression study by Prandi and colleagues identified Tas2r108, Tas2r126, and Tas2r135 being expressed in the mouse small intestine [10]. Theoretically, we would anticipate responses only to glycocholic acid and denatonium benzoate. Whereas the maximally employed glycocholic acid concentration of 100 µM just reaches the threshold concentrations for Tas2r108 and Tas2r126, the denatonium benzoate-activated Tas2r135 with a threshold concentration of 100 µM should become fully activated by 5 mM denatonium benzoate. This would explain the rather narrow response to the bitter stimulation paradigm employed in this study. We may assume that the absence of CCK secretion by stimulation with 500 µM denatonium benzoate could be due to a lower effective concentration of tastants at the receptor site in the complex tissue of organoids (e.g., caused by mucus secretion) or other modulatory effects, such as deviations in sodium concentration [51] or pH-values [52].

The activation of CCK secretion by 30 mM L-glutamic acid, but not by 3 mM or by the combined stimulation with 3 mM L-glutamic acid and IMP, suggests that the canonical umami receptor, Tas1r1/Tas1r3, which shows strong enhancement by IMP [25,53], is not involved in this process, but rather, one of the alternative umami receptors, truncated-mGluR1 [54] or truncated-mGluR4 [55], is involved in this process. Indeed, both alternative umami receptors have been proposed in the past and truncated-mGluR1 has been documented to be expressed in the murine stomach [56]. Whether the expression continues into the small intestine is, however, unclear.

Furthermore, the selective activation by bitter substances and L-glutamic acid suggests possible crosstalk between the canonical bitter (TAS2R/Tas2r) and amino acid-sensing (Tas1r1/Tas1r3 or CaSR) pathways in enteroendocrine cells, which could fine-tune hormone release according to luminal composition [11,57,58]. The shared calcium signaling node involved in bitter and amino acid sensing may allow stimulus-specific dynamics—such as rapid transients for bitter compounds versus more sustained responses for L-glutamic acid—to be translated into distinct hormone profiles (e.g., CCK). By integrating these responses in a fully differentiated 3D epithelium, our data strengthen the argument that mouse intestinal organoids are not only suitable for mechanistic genetics but also for modeling how gut-derived chemosensory signals influence systemic physiology. This positions MIOs as a scalable platform for future work on agonist-specific hormone regulation, gut–brain signaling, and the design of nutrition- or drug-based interventions targeting bitter-sensing enteroendocrine pathways.

## 5. Conclusions

In this study, we established mouse small intestinal tract organoids as a tool for investigating the physiological roles of taste receptors in this tissue. In doing so, we seek to overcome issues frequently associated with studies devoted to taste receptor functions in the GI tract, such as interindividual variations between mice, sampling of tissues from somewhat different locations, variable inclusion of adjacent cells, or the use of immortalized cell lines as a surrogate, by a comparatively stable model system. We documented not only the successful differentiation of the organoids into the various GI cell types, but also observed the release of CCK upon stimulation with distinct tastants. We believe this model system will significantly advance our research in this area.

## Figures and Tables

**Table 1:** List of the primary antibodies, secondary antibodies, and blocking peptide.

Epitope/Conjugated Group	Species/Type	Blocking Peptide	Product Numbers	Dilution
GLP-1	goat/polyclonal IgG	Yes	Santa Cruz (Heidelberg, Germany) (sc-7782)	1:1000
mucin-2	rabbit/polyclonal IgG	No	Santa Cruz (sc-15334)	1:50
chromogranin A	rabbit/polyclonal IgG	No	Immunostar (2BScientific GmbH, Haag an der Amper, Germany) (20086)	1:1000
villin	goat/polyclonal IgG	Yes	Santa Cruz (sc-7672)	1:200
lysozyme C	goat/polyclonal IgG	Yes	Santa Cruz (sc-27958)	1:1000
rabbit IgG/AlexaFluor 488	goat/IgG	No	Molecular Probes (Thermo Fisher Scientific, Waltham, MA, USA) (A11034)	1:2000
goat IgG/AlexaFluor 488	mouse/IgG	No	Molecular Probes (A10680)	1:2000

**Table 2:** Cell markers used for immunohistochemical staining of GI cell types.

GI Cell Type	Marker
Enterocyte	Villin
Goblet cell	Mucin-2
Enteroendocrine cell	Chromogranin A
Enteroendocrine L-cell	GLP-1
Paneth cell	Lysozyme

**Table 3:** Candidate mouse Tas2rs involved in bitter-stimulated CCK secretion of MIO based on published data [8,10,26].

Tas2r	Employed Bitter Agonist (Threshold Conc. (µM))	Confirmed Expression in Mouse Small Intestine	Ratio (Stimulus Conc./Threshold (Fold))
Tas2r105	Cycloheximide (0.01) cucurbitacin E (10) denatonium benzoate (100) glycocholic acid (300)		
Tas2r108	glycocholic acid (100)	Yes	0.1–1×
Tas2r114	cucurbitacin E (3)		
Tas2r117	glycocholic acid (3)		
Tas2r123	denatonium benzoate (300) glycocholic acid (30)		
Tas2r126	glycocholic acid (100)	Yes	0.1–1×
Tas2r135	denatonium benzoate (100)	Yes	5–50×
Tas2r140	denatonium benzoate (300)		
Tas2r144	denatonium benzoate (3000) glycocholic acid (100)		

## Data Availability

The original contributions presented in this study are included in the article. Further inquiries can be directed to the corresponding author.

## Abbreviations

The following abbreviations are used in this manuscript:

CCK	Cholecystokinin
DAPI	4′,6-Diamidino-2-phenylindole dihydrochloride
DMEM	Dulbecco’s modified Eagle medium
EGF	Epidermal growth factor
ELISA	Enzyme-linked immunosorbent assay
GLP-1	Glucagon-like peptide-1
HEPES	4-(2-hydroxyethyl)-1-piperazineethanesulfonic acid
HRP	Horseradish peroxidase
IMP	Inosine 5′-monophosphate
MIO	Mouse intestinal organoid
PBS	Phosphate-buffered saline
PFA	Paraformaldehyde
PYY	Peptide YY
SEM	Standard error of the mean
Tas2r/TAS2R	Taste 2 receptor
